# Influence of the Internalization Pathway on the Efficacy of siRNA Delivery by Cationic Fluorescent Nanodiamonds in the Ewing Sarcoma Cell Model

**DOI:** 10.1371/journal.pone.0052207

**Published:** 2012-12-20

**Authors:** Anna Alhaddad, Catherine Durieu, Géraldine Dantelle, Eric Le Cam, Claude Malvy, François Treussart, Jean-Rémi Bertrand

**Affiliations:** 1 Vectorologie et thérapeutiques anti-cancéreuses, CNRS UMR 8203, Université Paris Sud 11, Institut Gustave Roussy, Villejuif, France; 2 Signalisations, Noyaux et Innovations en Cancérologie, CNRS UMR 8126, Université Paris Sud 11, Institut Gustave Roussy, Villejuif, France; 3 Laboratoire de Physique de la Matière Condensée, CNRS UMR 7643, Ecole Polytechnique, Palaiseau, France; 4 Laboratoire de Photonique Quantique et Moléculaire, CNRS UMR 8537, Ecole Normale Supérieure de Cachan, Cachan, France; University of Quebect at Trois-Rivieres, Canada

## Abstract

Small interfering RNAs (siRNAs) are powerful tools commonly used for the specific inhibition of gene expression. However, vectorization is required to facilitate cell penetration and to prevent siRNA degradation by nucleases. We have shown that diamond nanocrystals coated with cationic polymer can be used to carry siRNAs into Ewing sarcoma cells, in which they remain traceable over long periods, due to their intrinsic stable fluorescence. We tested two cationic polymers, polyallylamine and polyethylenimine. The release of siRNA, accompanied by Ewing sarcoma EWS-Fli1 oncogene silencing, was observed only with polyethylenimine. We investigated cell penetration and found that the underlying mechanisms accounted for these differences in behavior. Using drugs selectively inhibiting particular pathways and a combination of fluorescence and electronic microscopy, we showed that siRNA gene silencing occurred only if the siRNA:cationic nanodiamond complex followed the macropinocytosis route. These results have potential implications for the design of efficient drug-delivery vectors.

## Introduction

Nanocarriers are promising tools for biology, because they provide a unique opportunity to overcome cellular barriers to improve the delivery of various molecules and drugs. However, their efficiency depends on the pathway by which they are internalized [1]. Several mechanisms have been implicated in the penetration of cells by nanocarriers. The precise mechanisms used depend on the physicochemical characteristics of these particles, such as their size, surface properties and chemical nature, and the nature of the target cells [1], [2].

Most nanocarrier internalization pathways involve either phagocytosis or endocytosis, which encompasses clathrin- and caveola-mediated endocytosis, macropinocytosis and other pathways [1]. It is difficult to study these pathways in detail, due to the lack of low-toxicity labels that can be traced over timescales of tens of hours. These requirements preclude, for example, the use of CdSe-based nanocrystal semiconductors (Quantum Dots), despite their unsurpassed brightness [3], due to the risk of toxic Cd^2+^ ion leakage [4]. In this context, fluorescent diamond nanocrystals (fNDs) are very attractive candidates for intracellular delivery. They have a perfectly stable intrinsic fluorescence (no bleaching and no blinking) [5], [6] and a high contrast in electron microscopy [7], which facilitates their sub-cellular localization. They are non toxic to various cell lines [8]–[10] and do not trigger any inflammatory response neither in cells at concentration 50 µg/ml [11], nor in mouse [12]. Finally, nanodiamond offer various possibilities for surface functionalization [13].

The fluorescence of fNDs arises from the embedded nitrogen-vacancy (NV) color centers that are created in high-pressure high-temperature type 1b synthetic diamonds, with emission in the red and near infrared regions of the spectrum [14]–[16]. NV color centers do not photobleach or blink. Thus, fNDs are highly suitable for studies requiring long-term traceability in cells cells [17] and small organisms [18].

We investigated the possible application of fNDs in drug delivery, using a Ewing sarcoma cell model. Ewing sarcoma is the second most common primary bone tumor, and this aggressive form of child cancer leads, in a quarter of cases, to detectable metastases, mostly in the lungs and bone marrow [19]. In 85% of cases of this cancer, the junction oncogene EWS-Fli1 is expressed, following a chromosomal translocation t(11,22). Its transcription leads to the production of the corresponding chimeric protein [20], [21]. The EWS-Fli1 protein modifies the regulation of various pathways involved in cell proliferation, differentiation and apoptosis [22], by acting on target genes [23], [24] and proteins, including growth factors, such as IGF1 [25] and VEGF-A [26]. The inhibition of EWS-Fli1 activity would therefore be an attractive therapeutic strategy, making it possible to stop cell proliferation and to restore apoptosis.

We previously reported the development of cationic fNDs to which it was possible to bind short interfering double-stranded RNAs (siRNA). The resulting particles interacted with the EWS-Fli1 messenger RNA, triggering its cleavage, thereby preventing the translation of this oncogene [27]. Both the polymers tested, polyallylamine (PAH) and polyethylenimine (PEI), contain primary amines and bind siRNA, but binding efficiency was found to be higher for PAH. We observed that the transfection efficiency depended on the nature of the cationic polymer used to coat the NDs. Preliminary results suggested that this effect might be due to a lower efficiency of siRNA release into the cells for ND-PAH than for ND-PEI, possibly reflecting the use of different internalization pathways by these two cationic nanoparticles. It has been shown, in the HeLa cell model, that naked (anionic) nanodiamonds (size 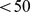 nm) are taken up by clathrin-dependent endocytosis [6], [9]. Similarly, dye-conjugated NDs penetrate neuronal cells (N2A cells) and enter intracellular vacuoles, including early endosomes and lysosomes [28]. In both cases, clathrin-mediated endocytosis is the major uptake pathway for naked fND.

In this study, we investigated the uptake mechanisms and cellular localization of the two different types of cationic polymer-coated fND vectors, using a combination of transmission electron microscopy (TEM), fluorescence confocal microscopy and assessments of the inhibition of EWS-Fli1 gene expression. We found that siRNA gene silencing occurred only if the siRNA:cationic nanodiamond complex followed the macropinocytosis route.

## Results

### Mechanisms of Cationic fND Uptake by Cells

#### Confocal microscopy

**Figure 1:**
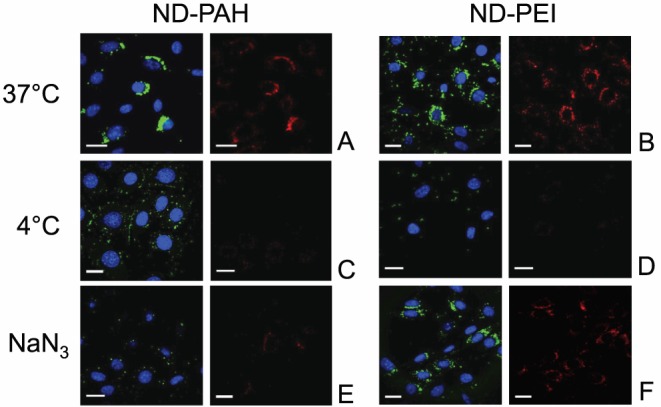
Study of the energy-dependent endocytosis of fluorescent nanodiamonds in NIH/3T3 EWS-Fli1 cells. The fNDs (red channel) loaded with FITC-labeled siRNA (green channel) were observed by confocal microscopy (Leica TSC SPE) after 4 h of incubation. We used 50 nM siRNA vectorized with ND-PAH or ND-PEI at mass ratios of 1∶25 and 1∶75, respectively. Cells were incubated at 37°C (control) (A, B), 4°C (C, D), or 37°C after treatment for 30 min treatment with NaN_3_ (E, F). Nuclei are labeled with DAPI (blue channel), scale bars: 20 µm.

We incubated fNDs-cationic polymer:siRNA conjugates for four hours with NIH/3T3 cells expressing the human EWS-Fli1 oncogene at 4°C or 37°C (control). After incubation at 4°C, a weaker photoluminescence signal from fNDs and siRNA-FITC was obtained for both ND-PAH and ND-PEI (Fig. 1C and D), than was observed after control incubation at 37°C (Fig. 1A and B), indicating a very low level of internalization of both types of complex. Interestingly, ATP depletion with NaN_3_, which should also lead to a blockade of endocytosis, entirely prevented the uptake of ND-PAH (Fig. 1E), but not that of ND-PEI (Fig. 1F).

**Figure 2:**
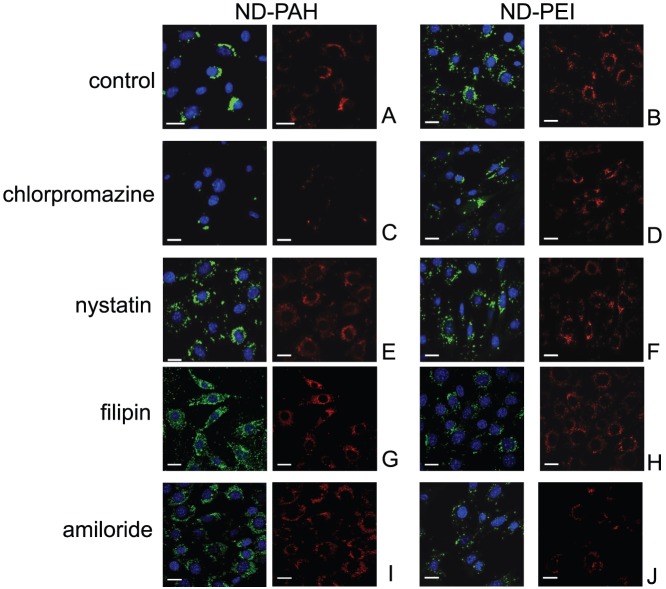
Study of the internalization pathways of siRNA-fND complexes in NIH/3T3 EWS-Fli1 cells, by fluorescence confocal microscopy. All the cells were incubated at 37°C, with drugs inhibiting specific internalization pathways: (C, D) chlorpromazine, blocking the clathrin pathway; (E, F) nystatin or (G, H) filipin, blocking the caveola pathway and (I, J) amiloride hydrochloride, inhibiting macropinocytosis. (A, B): control cells incubated with cationic ND-siRNA complexes. Red channel: fNDs; green channel: FITC-labeled siRNA. Blue channel: DAPI labeling. Observations were carried out after the cells had been incubated for 4 h with 50 nM siRNA vectorized with either ND-PAH (left columns) or ND-PEI (right columns), at mass ratios of 1∶25 and 1∶75, respectively. Scale bars: 20 µm.

Nevertheless, we considered endocytosis to be the most probable internalization mechanism. We therefore used selective inhibitors to determine whether the uptake process was mediated by clathrin or caveolae. The treatment of cells with chlorpromazine, an inhibitor of pit formation during the relocation of clathrin to the endosomes, reduced fND-PAH uptake strongly (Figure 2C), and fND-PEI uptake to a lesser extent (Figure 2D), as shown by comparisons with untreated cells. By contrast, blockade of the caveola pathway with filipin, which disrupts cholesterol synthesis, or with nystatin, which sequesters the cholesterol required for caveolin pit formation, had no effect on the uptake of either type of fND (Figure 2E–H). We also investigated the role of the macropinocytosis pathway, which has been shown to play an important role in the uptake of nanoparticles, particularly if they are aggregrated [29]–[31]. For this purpose, we treated the cells with amiloride, an inhibitor of Na^+^/H^+^ ATPase exchangers. We observed a partial inhibition of fND-PEI uptake, but not of fND-PAH uptake (Figure 2I–J). Thus, fND-PAH is internalized by clathrin-mediated endocytosis, whereas both clathrin-mediated endocytosis and macropinocytosis are involved in fND-PEI uptake.

#### Transmission electron microscopy

TEM observations were carried out on the same cells after 4 hours of incubation, for further investigation of the uptake mechanism and to visualize the subcellular localization of NDs coated with the two types of cationic polymers.

**Figure 3:**
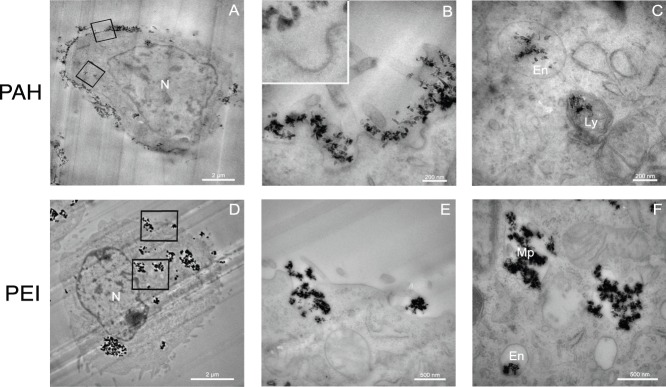
TEM observations of the distribution in NIH/3T3 EWS-Fli1 cells of ND-PAH (A)–(C) and ND-PEI (D)–(F) after 4 h of incubation. N: cell nucleus, En: endosomal compartment, Ly: lysosomal compartment, Mp: macropinosome compartment. (A) Most of ND-PAH are located on the outside of the cell membrane. (B) Higher magnification of the cell membrane (upper square-delimited region of (A)) displaying invaginations around aggregates of ND-PAH, an early stage in clathrin-mediated endocytosis. Inset: Higher magnification showing the clathrin pits, perpendicular to the invaginated membrane. (C) Magnification corresponding to the lower square-delimited region in (A), showing ND-PAH located in endosomal (En) and lysosomal (Ly) vesicles. (D) Most of the ND-PEI are present within the cell, in large structures in the perinuclear region. (E) Magnification corresponding to the upper square-delimited region in (D) showing pseudopods formed at the cell membrane during ND-PEI uptake. (F) Magnification corresponding to the lower square-delimited region in (D), showing ND-PEI localization, mostly in large macropinosome (Mp) vesicles, but also, to some extent, in endosomes (En). Scale bars in (A) and (D): 2 µm and in (B), (C), (E) and (F): 200 nm. TEM magnification in (A) and (D): ×4,400; in (B) and (C): ×50,000; in (E): ×20,000, and in (F): ×30,000.

Most of the ND-PAH remained outside the cell, forming aggregates at the cell surface 3A. Most of the internalized ND-PAH aggregates were present in endosome and lysosome vesicles (Figure 3A and C). Moreover, the classic shape of the clathrin-coated pits was visible in Figure 3B, confirming the conclusions drawn from fluorescence microscopy analysis.

**Figure 4:**
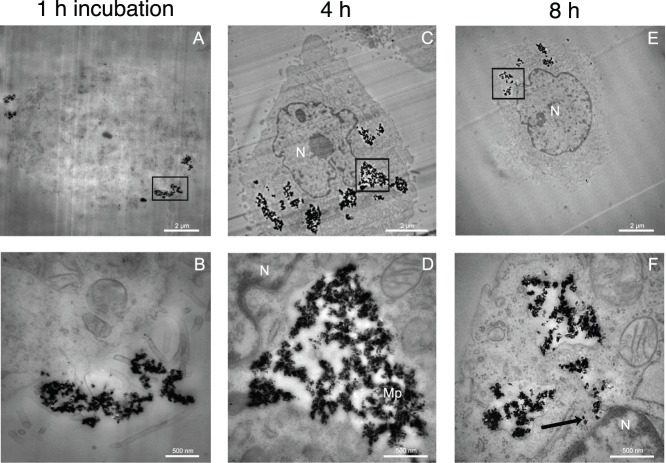
TEM imaging of the distribution of ND-PEI in NIH/3T3 EWS-Fli1 cells as a function of time N: cell nucleus, Mp: macropinosome compartment. The cell were incubated with ND-PEI for 1 h (A)&(B), 4 h (C)&(D) and 8 h (E)&(F). (A) ND-PEI were rapidly taken up by cells: at 1 h, some NDs had already crossed the cell membrane. (B), (D) and (F) are magnifications corresponding to the regions surrounded by rectangle or square in (A), (C) and (E) respectively. (B) Pseudopod formation at the cell membrane during ND-PEI uptake. At 4 h (C) and 8 h (E), all the ND-PEI are located in large perinuclear vacuoles within the cell. Most of these structures correspond to macropinosomes, which are still delimited by a membrane at 4 h (D), but are disrupted after 8 h of treatment, as confirmed by the presence of free diamond nanocrystals in the cytoplasm (arrow)(F). Scale bars in (A), (C) and (E): 2 µm and in (B), (D) and (F): 500 nm. TEM magnification in (A), (C) and (E): ×4,400; in (B): ×20,000; in (D) and (F): ×30,000.

By contrast to these observations for ND-PAH, ND-PEI were found exclusively within the cells after 4 hours of incubation. They appeared to be aggregated in the perinuclear region, in large vesicular structures (Figure 3D and Figure 4C). We also observed deformations of the membrane and the formation of pseudopods in the region of ND-PEI entry (Figure 3E). These structures facilitated the uptake of large ND aggregates by macropinocytosis. Most of the NDs in the cytoplasm were trapped in vacuoles with diameters of more than 500 nm (Figure 3F and Figure 4D). The TEM observations were therefore consistent with the findings of confocal microscopy studies, confirming the involvement of both macropinoctytosis and endocytosis in ND-PEI uptake.

We investigated the uptake kinetics of ND-PEI further, by performing TEM observations after various incubation times. After the incubation of ND-PEI with NIH/3T3 EWS-Fli1 cells for 1 h, most of the NDs were still located close to the cell membrane (Figure 4A). Some aggregates were also found at the external surface of the cell, surrounded by large deformations of the membrane in the form of pseudopodial extensions and endocytosis pits, indicative of the mechanisms preceding internalization (Figure 4B).

After 8 h of incubation, the distribution of ND-PEI within cells was similar to that after 4 h of incubation (Figure 4C–F), suggesting that this time period was sufficient for the completion of ND uptake. Figure 4 shows a similar location of ND-PEI within cells, in large vacuoles around the nucleus (Figure 4C and E). Nevertheless, the disruption of vacuolar membranes and the detection of some free ND-PEI in the cytoplasm (Figure 4F) suggest that some ND-PEI may have been released into the cytoplasm from these vacuoles before their fusion with the lysosomal compartment in which siRNA hydrolysis occurs.

### Inhibition of EWS-Fli1 Gene Expression by ND-PEI-vectorized siRNA in the Presence of Chemical Inhibitors of Internalization Pathways

**Figure 5:**
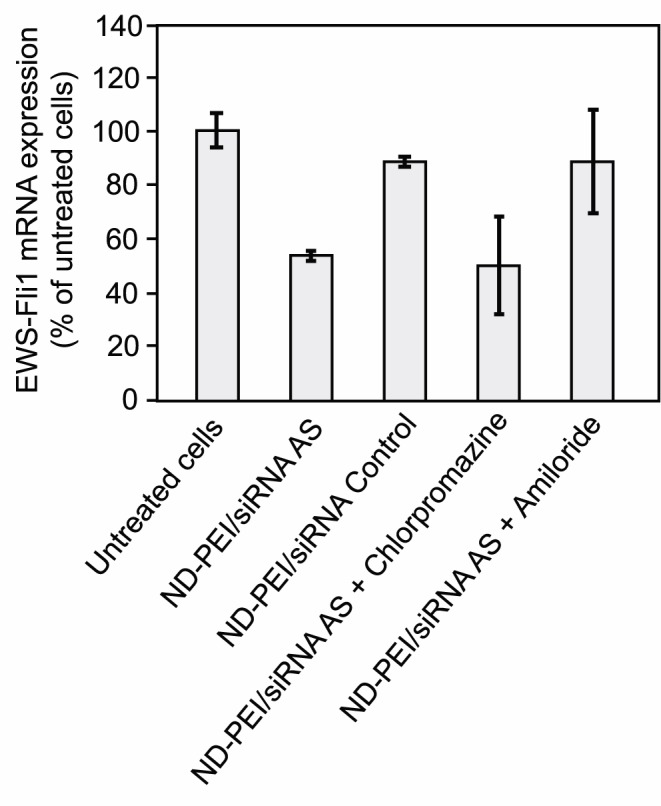
RT-qPCR analysis of EWS-Fli1 gene expression in NIH/3T3 EWS-Fli1 cells. The cells were treated with 50 nM antisense siRNA vectorized with ND-PEI at a mass ratio of 1∶75, with or without drugs specifically inhibiting particular internalization pathways: chlorpromazine, blocking the clathrin pathway and amiloride, inhibiting the macropinocytosis pathway. EWS-Fli1 gene expression is normalized with respect to that of the 18S rRNA gene.

We investigated the effect of the pathway of siRNA:ND-PEI uptake on the capacity of these particles to deliver siRNA efficiently to cells, by using quantitative RT-PCR to evaluate EWS-Fli1 gene expression in the presence of various pathway-specific chemical inhibitors. We found that siRNA:ND-PEI inhibited the expression of the target gene by 50% Figure 5. This effect was specific to the antisense siRNA, as treatment with a control siRNA had no effect. As a complentary study, we showed that siRNA:ND-PEI treatment of NIH-3T3 EWS-Fli1 cells results in an 2.7-fold increase of their apoptotic capabilities (Figure S1). Moreover, note that in the same conditions of concentration and mass ratio, the siRNA vectorized by PEI or PHA only (without the ND substrate) does not inhibit the mRNA expression (see Figure S2).

Following blockade of the clathrin-dependent endocytosis pathway by chloropromazine treatment, we observed a similar 50% inhibition of EWS-Fli1 gene expression, indicating that this pathway was not responsible for siRNA release and the subsequent specific degradation of the target mRNA in cells. By contrast, no inhibition of expression (absolute values of only 3% inhibition) was observed when the cells were treated with amiloride to inhibit the macropinocytosis pathway. This indicates that macropinocytosis is the siRNA:ND-PEI uptake pathway leading to specific mRNA degradation in cells.

## Discussion

We previously showed that uncoated 46 nm-diameter fNDs prepared as described in the Materials and Methods were taken up via clathrin-mediated endocytosis in HeLa cells [7]. This pathway is the internalization pathway most commonly used by nanoparticles in this size range [32].

We show here that a cationic polymer coating can change the internalization mechanism used, potentially leading to the efficient release of siRNA and specific mRNA inhibition. Clathrin-mediated endocytosis remained the predominant internalization pathway for the siRNA:ND-PAH complex. This was confirmed by TEM observations showing clathrin pits of 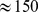 nm in size at the cell surface (inset Figure 3B). This pits are an assembly of basket-like structures formed by the polymerization of clathrin units [33]. By contrast, siRNA:ND-PEI was taken up via two pathways: clathrin-mediated endocytosis and macropynocytosis (Figure 2).

Macropynocytosis is the preferred pathway for the uptake of particles of 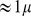
m in size, such as aggregates of nanoparticles. It involves the formation of actin-driven membrane protrusions, which collapse and fuse with the plasma membrane [34], trapping the particles in large vesicles called macropinosomes (Fig. 3F and Fig. 4D). These vesicles may eventually fuse with lysosomes or recycle their content to the surface [35]. Use of the PEI vector also prevents the lysosomes from becoming acidic, due to its buffering capacity (proton sponge-effect [36], [37]), which manifests as an osmotic swelling of the aqueous medium, eventually leading to vacuole disruption (Figure 4F). This phenomenon, which was observed at longer incubation times (8 h) may account for the greater efficiency of siRNA vectorized with ND-PEI than of siRNA vectorized with ND-PAH. We also noticed that after incubation for 4 h, all the ND-PEI were internalized (Fig. 3A), whereas a large proportion of the ND-PAH remained on the outer surface of the cell (Fig. 3D). This provides an additional explanation for ND-PEI vector greater efficacy.

siRNA:ND-PAH was initially taken up into early endosomes (internal pH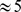), which then fused with lysosomes (Figure 3C), which have a harsh internal environment, favoring the degradation of their contents. For biological activity, the siRNA must be released from the nanodiamonds before lysosomal degradation [38]. However, the detection of ND-PAH in lysosomes and the perfect colocalization of the siRNA with ND-PAH for up to 72 h after internalization, as demonstrated in a previous study [27], indicate that the siRNA is probably degraded within the lysosome, precluding any effect on the target mRNA.

Both clathrin-dependent endocytosis and macropinocytosis were involved in the uptake of ND-PEI. However, assessments of the silencing activity of ND-PEI-vectorized siRNA in the presence of various specific pathway inhibitors clearly showed that only the macropinocytosis inhibitor blocked the effects of the siRNA (Figure 5). Thus, macropinocytosis was the only route that delivered the active siRNA to cells, inducing degradation of the target mRNA.

We also investigated whether the size of the internalized complex determined the internalization pathway used. As stated above, clathrin-dependent endocytosis is the predominant pathway for small particles, whereas larger particles tend to be taken up by macropinocytosis [32]. ND-PEI displayed a much higher degree of aggregation than ND-PAH in the culture medium and at the cell surface. We therefore cannot exclude the possibility that macropinocytosis occurred simply due to a size effect consecutive to the formation of large ND-PEI aggregates. We tested this hypothesis, by studying the internalization of ND-PAH with a primary size of ≲**≲**200 nm, to determine whether uptake by macropinocytosis uptake was entirely dependent on size. TEM imaging revealed very low levels of uptake for these larger ND-PAH (Figure S3). Thus, the primary size of diamond particle is not the determining factor of the pathway. However, the size and the shape of nanodiamond aggregates are dynamical parameters that may also influence the internalization route. “Dynamical aggregates”, defined as aggregates of nanoparticles with weak attractive interactions allowing changes of shape, present to the cell membrane a large surface of interaction at the early stages of internalization that may favor macro-pinocytosis instead of endocytosis. The validation of this explanation would require measurements of ND-PEI and ND-PAH aggregates comparative stability in serum supplemented culture medium, which is beyond the scope of this study.

In conclusion, we have shown that the nature of the polymer coating plays an essential role determining the siRNA:ND uptake mechanisms used and the biological activity of the siRNA. ND-PEI internalization occurred by both endocytosis and macropinocytosis, whereas ND-PAH tended to stay outside the cell, entering only by clathrin-mediated endocytosis. Despite the existence of several different pathways for cationic ND uptake, our results indicate that only the macropinocytosis pathway, which mediates the uptake of ND-PEI:siRNA, allows the efficient delivery of siRNA to the cell, resulting in destruction of the target mRNA. By contrast, the clathrin-dependent endocytosis pathway delivers the siRNA and its carrier to the lysosomal compartment, in which siRNA hydrolysis probably occurs. All these results have potential implications for the design of new solid state-based drug-delivery vehicles.

## Materials and Methods

### Coating of Nanodiamonds with Cationic Polymers

This procedure was initially optimized with non fluorescent nanodiamonds with a mean size of 50 nm, as received from the manufacturer (SYP 0–0.05, 50 nm; VanMoppes, Geneva, Switzerland). These particles are cleaned in a highly oxidative acid mixture by the manufacturer and their dispersion in water yields a colloidal suspension with negative zeta potential values (−28 mV) [27], attributed to the carboxyl groups on the surface of the diamond.

Anionic NDs were dispersed in deionized water at a concentration of 1 g.L^−1^. This suspension was sonicated for 3 h, at 300 W (Vibra-Cell with a 3 mm stepped microtip) to ensure that aggregates were disrupted.

We coated the nanodiamonds with branched low-molecular weight (800 Da) polyethylenimine (PEI, Sigma-Aldrich Ref. 408719) and polyallylamine hydrochloride (PAH, Sigma Aldrich Ref.283215), by adding the 1 g.L^−1^ suspension of NDs in water dropwise to the PAH solution (v/v) (1 g.L^−1^, 3 mM NaCl) or to the PEI solution (at the ND particle: PEI molar ratio was 1∶10, as in the study by Zhang et al. [39]) and then sonicating at 300 W, for 15 min. The mixtures were stirred overnight at room temperature, then washed three times with deionized water and centrifuged at 30,000 rpm for 3 h (Optima XL90 Ultracentrifuge, 50Ti rotor, Beckman Coulter Inc., USA). The ND-polymer pellet was resuspended in water, and the concentration of the suspension was determined by freeze-drying and weighing 1 ml of the suspension. The cationic polymer-coated diamond nanocrystals obtained had a mean size of 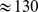 nm for ND-PAH, and 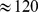 nm for ND-PEI [27].

### Cell Culture

All experiments were performed in NIH/3T3 murine fibroblasts stably transfected with the human EWS-Fli1 oncogene, which was generously provided by Dr J. Ghysdael (CNRS UMR 3306, Institut Curie, Orsay, France). This modified cell line has already been used in various experiments by different teams (e.g. Ref. [40] and [41]). Briefly, these cells are NIH/3T3 fibroblasts purchased from ATCC (CRL1658) that have been stably transduced with a cDNA encoding the type 1 EWS-Fli1 fusion protein gene inserted downstream from the Mo-MuLV long terminal repeat in the pBABE-puro retroviral vector. All the NIH/3T3-derived cell lines were grown at 37°C, under a moist atmosphere containing 5% CO_2_, in DMEM (Gibco®, Invitrogen Corp., USA) supplemented with 10% newborn calf serum (Gibco), 1% penicillin-streptomycin (Gibco). NIH/3T3 EWS-Fli1-derived cells were selected on medium containing 2.5 µg/mL puromycin (Sigma). The cells were used between passages 2 and 12 after thawing, to avoid problems due to phenotypic instability.

### Cell Transfection with Vectorized siRNA

Cell were transfected with siRNA following the physical adsorption to the cell surface of ND-PAH or ND-PEI. The siRNA sequences were designed to target the oncogenic EWS-Fli1 junction in the chimeric mRNA (nucleotides 822–842). We purchased siRNAs with the following sequences from Eurogentec (Lièges, Belgium): Antisense siRNA, sense strand: 5-r(GCUACGGGCAGCAGAACCC) d(TT)-3 and antisense strand: 5-r(GGGUUCUGCUGCCCGUAGC) d(TG)-3; Control siRNA, sense strand: 5-r(GCCAGUGUCACCGUCAAGG) d(AG)-3 and antisense strand: 5-r(CCUUGACGGUGACACUGGC) d(TT)-3.

The sense and antisense strands were hybridized at a concentration of 20 µM in annealing buffer (Eurogentec), by heating for 5 min at 95°C and then for 1 h at 37°C. NIH/3T3 EWS-Fli1 cells were used to seed 12-well plates at a density of 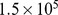 cells per well in 500 µl of the corresponding medium 24 h before transfection. Complexes of ND with antisense and control siRNAs were prepared as follows. The siRNAs were prepared at a concentration of 250 nM in 100 mM NaCl, 10 mM Hepes buffer at pH 7.3. A volume of 40 µl of the resulting solution was mixed with 60 µl of ND-PAH or ND-PEI prepared in 100 mM NaCl, 10 mM Hepes buffer pH 7.3, at concentrations corresponding to 1∶25 and 1∶75 mass ratios of siRNA:ND-PAH and siRNA:ND-PEI, respectively, as determined in a previous study [27].

The mixture was incubated for 15 minutes at room temperature, to allow ND:siRNA complexes to form. The medium in each well was replaced with 400 µl of fresh culture medium supplemented with serum and 100 µl of ND:siRNA complex. The final concentrations in each well were 50 nM siRNA, and 48.8 µg/ml ND-PEI or 16.27 µg/ml ND-PAH. The cells were then incubated for the times indicated.

### Study of the Pathway Used for Nanodiamond Internalization

We used fluorescent nanodiamonds (fNDs) to monitor the presence of siRNA vectors within cells. The fNDs were prepared as described by Dantelle et al. [14]. Briefly, high-pressure high-temperature (HPHT) nanodiamonds, purchased from Van Moppe, were irradiated with a 13.9 MeV electron beam for 1 hour, to create vacancies. Irradiated nanodiamonds were then annealed for 2 hours under a vacuum, to stabilize the vacancy (V) next to a nitrogen (N) impurity, to form fluorescent NV color centers, which emit in the far-red and near-infrared regions of the spectrum (600–750 nm). We removed the graphite that formed on the surface of the fND that would otherwise have quenched fluorescence [42], by oxidizing the powder at 550°C in air for 2 hours. It was then dispersed in deionized distilled water (DDW) and sonicated for a few minutes, yielding a stable colloidal suspension of nanodiamonds, which were then coated with cationic polymers, as described above, to generate fND-PAH and fND-PEI.

The cells were plated on 18 mm diameter coverslips, in 12-well dishes (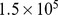 cells, in a volume of 500 µl of culture medium per well, one coverslip per well) and allowed to grow for 24 h. The fND-PEI or fND-PAH were complexed with fluorescently labeled siRNA (3-FITC-antsense siRNA, Eurogentec) at a final concentration of 50 nM in DDW, at an siRNA:fND weight ratio of 1∶25 for ND-PAH and 1∶75 for ND-PEI. We added various chemical inhibitors of internalization (see below), as required, 30 min before cell transfection. The culture medium was then replaced with 100 µl of siRNA:fND complexes in 10 mM HEPES buffer pH 7.3, 100 mM NaCl and the final volume was made up to 500 µl with DMEM supplemented with 10% FCS (Gibco) and the corresponding chemical inhibitor. After 4 h of incubation, cells were washed twice with PBS, fixed by incubation with 4% paraformaldehyde in PBS solution for 20 min at room temperature, washed three times with PBS and mounted in DAPI Fluoromount G (Southern Biotech, Birmingham, AL, USA). Fixed cells were observed by confocal microscopy (TCS SPE Leica). The green detection channel corresponded to the 501–543 nm wavelength range, and was used to assess FITC emission, whereas the red channel was associated with the 654–702 nm wavelength range and was used for the detection of NV fluorescence centers.

#### Selective inhibition of internalization pathways with drugs

Endocytosis blockade: endocytosis is an energy-dependent process and can therefore be blocked in two ways: low-temperature incubation or ATP depletion. For low-temperature inhibition, the cells were grown as described above and incubated for 4 h at 4°C or at 37°C. For ATP depletion, we added 1 mg/ml NaN_3_ (Sigma-Aldrich) and incubated the mixture at 37°C.

For the selective blockade of clathrin-dependent endocytosis, we used 10 µg/ml chlorpromazine hydrochloride (Sigma-Aldrich), with incubation at 37°C.

For the selective blockade of caveola-dependent endocytosis, the cells were treated with 5 µg/ml filipin (Sigma-Aldrich) or 50 µg/ml yystatin (Sigma Aldrich) at 37°C.

For the selective blockade of macropinocytosis, the cells were treated with 100 µM amiloride hydrochloride (Sigma-Aldrich) at 37°C. All these treatments were performed 30 min before the addition of siRNA-ND complexes.

### Transmission Electron Microscopy (TEM)

Samples for TEM imaging were prepared as follows. NIH/3T3 EWS-Fli1 cells were used to seed plates, for 24 h, in the standard conditions. NDs were added to the cell culture medium at concentrations of 48.8 µg/ml for ND-PE and, 16.27 µg/ml for ND-PAH, and the resulting suspensions were incubated for 1 h, 4 h or 8 h at 37°C. Adherent and floating cells were then harvested from the culture, centrifuged [1], and the cell pellets were fixed by incubation for 1 hour at room temperature in 2% glutaraldehyde (EMS, Hatfield, PA, USA) in 0.1 M cacodylate buffer pH 7.4.

Cell pellets were post-fixed by incubation for 1 hour at room temperature with 1% osmium tetroxide (EMS, Hatfield, PA, USA) in 0.1 M cacodylate pH 7.4 buffer and 1.5% potassium ferrocyanide (Sigma-Aldrich, France). They were dehydrated by adding increasing concentrations of ethanol and were then embedded in Epon 812 epoxy resin (EMS, Hatfield, PA, USA). The resin was polymerized by heating for 72 hours at 56°C.

It was then sectioned with a microtome (sections between 90 and 200 nm thick), and the sections were collected on collodion-carbon-coated copper grids. We added uranyl acetate (Merck, France) and lead citrate solutions (Reynold’s stain) to the sections to improve the contrast of TEM cell images. The sections were observed with a Zeiss 902 TEM in bright-field conditions and in the filtered zero-loss mode, with a CCD array detector (Megaview III, Olympus) coupled to SIS software (Olympus).

### Real-time Quantitative RT-PCR

Total RNA was extracted as follows. The cells were incubated for 24 h, washed with PBS and lysed with 800 µl TRIzol, (Invitrogen, France). we then added 160 µl chloroform, vortexed, and centrifuged the mixture at 13,000 rpm [2] for 15 minutes. We then mixed 300–350 µl of the aqueous phase with an equal volume of isopropanol, incubated the mixture for 15 min at room temperature and then centrifuged it at 13,000 rpm [3] for 15 min at 4°C. The pellet was washed twice with 70% ethanol, dried at room temperature and then dissolved in 10 µl of water supplemented with 0.5 U/µl RNasin (Promega). Total RNA was quantified by spectrophotometry (UV-1605, Shimadzu) at a wavelength of 260 nm.

Reverse transcription was performed on 1.5 µg of total RNA by adding 2 µl of random hexamers at a concentration of 50 µg/ml (Promega), and heating at 65°C for 5 min. The RNA was then incubated with 0.5 µl M-mLV reverse transcriptase 200 U/µl, 0.5 µl 20 mM DNTP, 0.5 µl RNasin (40 U/µl) and 4 µl of 5× Buffer (Promega) for 1 h at 42°C.

PCR quantification was carried out with QPCR SuperMix SYBR GreenER (Invitrogen, France). The EWS-Fli 1 gene was amplified with the EWS- Forward Primer: 5-AGC AGT TAC TCT CAG CAG AAC ACC-3 and Fli 1-reverse primer: 5-CCA GGA TCT GAT ACG GAT CTG GCT G-3 (Eurogentec). We mixed 1 µl of each primer, at a concentration of 10 µM, with 5 µl of cDNA diluted 1/20 (v/v) in a final volume of 25 µl. Samples were amplified over 45 cycles, in a 7900 Fast Real-Time PCR System (Applied Biosystems), as follows: 2 minutes of incubation at 50°C, 10 min at 95°C, followed by 45 cycles of 95°C for 15 seconds, 60°C for 1 min. The human 18S rRNA gene was used as a control and was amplified with the 18S Forward Primer 5-CGT TCA GCC ACC CGA GAT-3, and 18S reverse primer 5 TAA TGA TCC TTC CGC AGG TT-3. The Ct obtained was between 10 and 16 for 18S and between 20 and 24 for EWS-Fli1. Comparative Ct methods were used to normalize the target Ct by the 18S control gene Ct.

## Supporting Information

Figure S1 **Flow cytometer measurement of apoptotic cell population (Anexin V^+^/Propidiume Iodide^−^) after treatment of NIH/3T3 EWS-Fli1 cells by various compounds.** Only the treatment by the AntiSens (AS) siRNA directed against EWS-Fli1 oncogene and vectorized by ND-PEI leads to an increase in apoptotic cell number. siRNA Ct is the 18S siRNA sequence.
(EPS)

Figure S2 **RT-qPCR measurement of EWS-Fli1 mRNA targeted by siRNA with AntiSens (AS) action in NIH/3T3 EWS-Fli1 cells.** siRNA is vectorized by either cationic ND (ND-PAH or ND-PEI) or free polycations (PAH or PEI). siRNA:ND-PEI promotes 40% inhibition of mRNA expression while free PEI and free PAH have no inhibiting effect.
(EPS)

Figure S3 **TEM observations of ND-PAH of size ≲≲200 nm in NIH/3T3 EWS-Fli1 cells after 4 h of incubation.** Almost no ND-PAH are uptaken by the cells (A). A few nanocrystals are observed at the surface (B) and some are detected in lysosomal and endosomal compartments (C). Scale bars in (A): 2 µm and in (B) and (C): 500 nm. TEM magnification in (A): ×4,400, and in (B) and (C): ×50,000.
(EPS)

Text S1 **This file contains three sections: section 1 on the “Incidence of EWS-Fli1 inhibition by siRNA:polycationic ND on apoptotic status of NIH/3T3 EWS-Fli1 cells”, section 2 on the “Comparison of siRNA inhibition of EWS-Fli1 expression in NIH/3T3 EWS-Fli1 cells when it is vectorized by free PEI (PAH), or ND-PEI (resp. ND-PAH)”, and finally section 3 on the “Distribution of ≲≲200 nm ND-PAH nanoparticles as observed by TEM”.** Figures S1, S2 and S3 cited in this text file are available as separate files.
(PDF)
