# Supplementary material for: Influence of the Internalization Pathway on the Efficacy of siRNA Delivery by Cationic Fluorescent Nanodiamonds in the Ewing Sarcoma Cell Model
Source: PLoS One. 2012 Dec 20;7(12):e52207. doi: 10.1371/journal.pone.0052207 (PMC3527409; doi:10.1371/journal.pone.0052207)
Supplement: Text S1 — This file contains three sections: section 1 on the “Incidence of EWS-Fli1 inhibition by siRNA:polycationic ND on apoptotic status of NIH/3T3 EWS-Fli1 cells”, section 2 on the “Comparison of siRNA inhibition of EWS-Fli1 expression in NIH/3T3 EWS-Fli1 cells when it is vectorized by free PEI (PAH), or ND-PEI (resp. ND-PAH)”, and finally section 3 on the “Distribution of ≲≲200 nm ND-PAH nanoparticles as observed by TEM”. Figures S1, S2 and S3 cited in this text file are available as separate files. (PDF) [file pone.0052207.s004.pdf]

Supporting information

"Influence of the internalization pathway on the efficacy of siRNAs delivery by cationic fluorescent nanodiamonds in Ewing sarcoma model cells"

Anna Alhaddad, Catherine Durieu, Géraldine Dantelle, Eric Le Cam, Claude Malvy, François Treussart and Jean-Rémi Bertrand

1 Incidence of EWS-Fli1 inhibition by siRNA:polycationic ND on apoptotic status of NIH/3T3 EWS-Fli1 cells.

The expression of EWS-Fli1 oncogene in cell is described as an early event in cancerous cell transformation. This oncogene modulates many regulatory pathways such as IGF1 growth factor pathway [1] and inhibition of apoptosis involving p21 protein [2]. In this context, we may expect that inhibition of EWS-Fli1 restores a functional apoptosis into cell and then cell sensitivity to chemotherapy.

The apoptotic status of NIH/3T3 EWS-Fli1 was tested after siRNA treatment by the annexin V methods. The cells were treated for 72 h with 50 nM of free siRNA (control) or siRNA:polycationic ND as described in Materials and Methods. After annexin V (ApopNexin Annexin V FITC Apoptosis Kit, EMD Millipore Corp., USA) and propidium iodine (PI) coloration, the cells are analysed by flow cytometry (Accuri C6, BD Biosciences, USA). Apoptosis cells fixed the annexin V-fluorescein (green fluorescence) but have intact membrane and are not permeant to PI (red fluorescence). Apoptotic cells therefore correspond to anexin V^{+}/PI^{-} population, which is displayed in Figure S1 taking cells not treated with siRNA:polycationic ND as the reference.

We observe that free siRNA and the two types of cationic ND alone do not induce a modification of apoptotic cell number, as well as the 18S siRNA control sequence (siRNA Ct) vectorized by either ND-PEI or ND-PAH. On the other hand, when the cells are treated by siRNA AntiSens:ND-PEI there is a 2.7-fold increase of apoptotic cell number, in agreement with the inhibition of EWS-Fli1 expression (Ref. [3] and Figure S2) and associated cell proliferation. siRNA:ND-PEI partially restore the capabilities of NIH/3T3 EWS-Fli1 cells to enter in apoptosis. This result indicates that siRNA directed against EWS-Fli1 oncogene and efficiently vectorized by NE-PEI could potentiate the action of classical chemotherapeutic agent.

2 Comparison of siRNA inhibition of EWS-Fli1 expression in NIH/3T3 EWS-Fli1 cells when it is vectorized by free PEI (PAH), or ND-PEI (resp. ND-PAH).

It is well known that cationic polymer such as PEI are efficient vectors for nucleic acids (including siRNA) delivery to cells [4]. Thus, the siRNA inhibitory effect we observed could be the result of the polycations detaching from the ND, then encapsulating the siRNA, and finally triggering its internalization. To check if such a scenario takes place, we have compared in the same experiment, the ability of free and ND-bound PEI and PAH to vectorise efficiently the siRNA and then promote EWS-Fli1 silencing in cells.

In the case of siRNA associated to ND-PAH or ND-PEI, we used the optimal siRNA:ND-polycation mass ratio of 1:25 and 1:75 respectively (siRNA concentration: 50 nM) as determined in our previous work [3], corresponding to masses $m_{\rm ND-PAH} = 4.07\,\mu$g/well (well volume: 500 μl) for ND-PAH and $m_{\rm ND-PEI} = 12.2\,\mu$g/well for ND-PEI. To form the siRNA:PAH and siRNA:PEI complexes, we used $m_{\rm PAH} = 0.23\,\mu$g/well for the PAH and $m_{\rm PAH} = 0.0145\,\mu$g/well for the PEI. Higher concentrations of free polycations, resulted in cyto-toxicity, with still no mRNA expression inhibition effect.

After cell treatment for 24 hours, EWS-Fli1 expression in NIH/3T3 EWS-Fli1 cell is determined by RT-qPCR as described in the Materials and Methods. Figure S2 shows that ND-PEI vectorized siRNA promote 40% inhibition of EWS-Fli1 mRNA expression, while no significant inhibition is observed for siRNA vectorised by ND-PAH as previously reported [3], neither for siRNA vectorised by free PEI or PAH. These results are confirmed at the protein level by western blotting analysis (data not shown). The inefficiency of free PEI to vectorise siRNA in cell may be due to the use of low molecular weight polymer (800 Da) as reported by Pereira et al. [5] who observed that siRNA:PEI (1300 Da) polyplex is not stable in physiological conditions contrary to siRNA:PEI (10,000 Da).

3 Distribution of $\lesssim 200$ nm ND-PAH nanoparticles as observed by TEM

In order to evaluate the role of the particle size in the internalization pathway followed, we prepared ND-PAH with size $\lesssim$ 200 nm diamond nanocrystlals similar to the size of the aggregates of 50 nm

ND (Fig. 3C). After 4 h of incubation of these cationic nanodiamonds with NIH/3T3 EWS-Fli1 cells, we detect only a few nanocrystals in lysosomal and endosomal compartments (Figure S3), while the majority are still in the culture medium and a few are located at the cell surface. Therefore, the use of diamond nanocrystals larger than 50 nm resulted in a smaller internalization yield, and cannot account for the triggering of the macropynocytosis pathway.

References

1. Prieur A, Tirode F, Cohen P, Delattre O (2004) EWS/FLI-1 silencing and gene profiling of Ewing cells reveal downstream oncogenic pathways and a crucial role for repression of insulin-like growth factor binding protein 3. Mol Cell Biol 24: 7275–7283.

2. Maitra A, Roberts H, Weinberg AG, Geradts J (2001) Aberrant expression of tumor suppressor proteins in the Ewing family of tumors. Arch Pathol Lab Med 125: 1207–1212.

3. Alhaddad A, Adam MP, Botsoa J, Dantelle G, Perruchas S, et al. (2011) Nanodiamond as a Vector for siRNA Delivery to Ewing Sarcoma Cells. Small 7: 3087–3095.

4. Boussif O, Lezoualch F, Zanta MA, Mergny MD, Scherman D, et al. (1995) A Versatile Vector for Gene and Oligonucleotide Transfer Into Cells in Culture and in-Vivo - Polyethylenimine. P Natl Acad Sci Usa 92: 7297–7301.

5. Pereira P, Jorge AF, Martins R, Pais AACC, Sousa F, et al. (2012) Characterization of polyplexes involving small RNA. Journal of Colloid and Interface Science 387: 84–94.

⋆ ⋆ ⋆
